# Shesher and Welala Floodplain Wetlands (Lake Tana, Ethiopia): Are They Important Breeding Habitats for *Clarias gariepinus* and the Migratory *Labeobarbus* Fish Species?

**DOI:** 10.1100/2012/298742

**Published:** 2012-04-30

**Authors:** Wassie Anteneh, Eshete Dejen, Abebe Getahun

**Affiliations:** ^1^Department of Biology, College of Science, Bahir Dar University, P.O. Box 79, Bahir Dar, Ethiopia; ^2^FAO-Sub Regional Office for Eastern Africa, P.O. Box 5536, Addis Ababa, Ethiopia; ^3^Fisheries and Aquatic Science Stream, Faculty of Life Sciences, Addis Ababa University, P.O. Box 1176, Addis Ababa, Ethiopia

## Abstract

This study aims at investigating the spawning migration of the endemic *Labeobarbus* species and *C. gariepinus* from Lake Tana, through Ribb River, to Welala and Shesher wetlands. The study was conducted during peak spawning months (July to October, 2010). Fish were collected through overnight gillnet settings. A total of 1725 specimens of the genus *Labeobarbus* (13 species) and 506 specimens of *C. gariepinus* were collected. Six species of *Labeobarbus* formed prespawning aggregation at Ribb River mouth. However, no *Labeobarbus* species was found to spawn in the two wetlands. More than 90% of the catch in Welala and Shesher wetlands was contributed by *C. gariepinus*. This implies that these wetlands are ideal spawning and nursery habitats for *C. gariepinus* but not for the endemic *Labeobarbus* species. Except *L. intermedius*, all the six *Labeobarbus* species (aggregated at Ribb River mouth) and *C. gariepinus* (spawning at Shesher and Welala wetlands) were temporally segregated.

## 1. Introduction

The contemporary *Labeobarbus *species of Lake Tana (Ethiopia) form the only known remaining intact species flock of large cyprinid fishes, since the one in Lake Lanao in the Philippines has almost disappeared due to destructive fishing [1]. The vast majority of cyprinids occur in rivers, but some *Labeobarbus* and *Labeo* species are adapted to a lacustrine environment [2]. However, these lake-dwelling cyprinids spawn in rivers, by undertaking a single annual breeding migration up rivers [3]. This spawning strategy makes the large African cyprinids vulnerable for modern fisheries, since the fishermen target spawning aggregations at river mouths by effectively blocking them off from the lake, preventing mature individuals from reaching the upstream spawning areas [4, 5]. Although the causes for the decline are not properly identified, the migratory riverine spawning species of *Labeobarbus* in Lake Tana have undergone drastic decline (>75% in biomass and 80% in number) from 1991 to 2001 [6, 7].

The other commercially important species in Lake Tana, *C. gariepinus *[8], at the beginning of the rainy season (June-July), moves through the littoral areas towards the inundated floodplains and upstream inflowing rivers for spawning [7, 8]. *Clarias gariepinus *is the most dominant species during the rainy season upstream of the turbid Ribb River probably due to the availability of extended floodplain [9]. When the water level starts to decrease (October–December), *C. gariepinus *migrates back through the littoral zone towards the pelagic zone (Lake Tana). *Clarias gariepinus *is targeted by the commercial gillnet fishery when migrating between the floodplains (spawning areas) and the lake [7]. Although the large, older individuals proved to be vulnerable for increased mortality by the commercial gillnet fishery, it is known that, compared with *Labeobarbus* spp.*, C. gariepinus *is only moderately susceptible to fishing pressure in Lake Tana. This is because *C. gariepinus* is found to be more resilient [10]. In the last decades, as a result of the low monetary value and poor preference to this species by the Ethiopians, it was not selectively targeted by the commercial gillnet fishery and is mainly landed as bycatch [7]. However, according to Atnafu [11], *C. gariepinus* recently has become highly preferred fish by the commercial fishermen in Lake Tana area for dry fish export, especially to Sudan.

Various studies [9, 12–17] showed that seven (*L. macrophthalmus, L. truttiformis, L. megastoma, L. brevicephalus, L. tsanensis, L. platydorsus, *and *L. acutirostris*) of the 15 endemic *Labeobarbus* species migrate more than 50 km up rivers during the rainy season to spawn in fast flowing, clear and gravel bed streams. However, mass spawning migrations for the remaining eight *Labeobarbus* species (*L. nedgia, L. dainellii, L. gorguari, L. longissimus, L. intermedius, L. gorgorensis, L. surkis, *and *L. crassibarbis*) were missing from all tributaries studied so far. According to de Graaf et al. [14], these missing species may spawn in the lake or adjacent floodplain wetlands.

**Figure 1:**
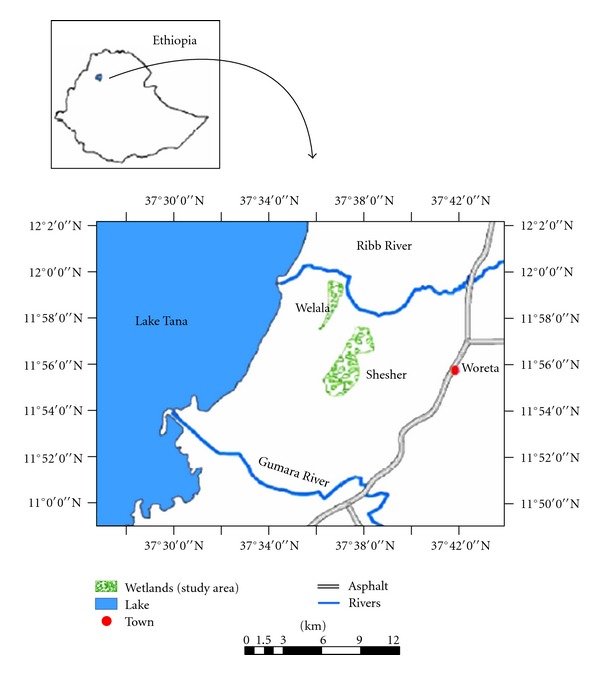
Map of Lake Tana and Ribb River and associated Shesher and Welala floodplain wetlands (after Atnafu et al. [18]).

The Shesher and Welala wetlands are located 3–5 km away from Lake Tana (Figure 1) and are valuable for the local community. They provide fishes, water, and grazing for livestock. They also harbor large diversity of bird species including internationally endangered and threatened ones [18]. They are the buffering zones of Lake Tana [17]. However, due to unsustainable farming activities by local farmers, the existence of these floodplain wetlands and associated ecological services as well as socioeconomic importance is under threat [18, 19]. It was observed that the local farmers were draining and pumping the water to expand farming land. Another potential threat is the large dam under construction on Ribb River that could minimize the water overflowing to these wetlands [20]. To have management plans for the two wetlands and also to conduct environmental impact assessment studies for all future development projects around the Lake Tana are strongly recommended.

Probably due to remoteness and inaccessibility, data about the ecological importance of the two prominent wetlands, Welala and Shesher, for the migratory fishes of Lake Tana are totally absent. Ribb River charges these wetlands as it overflows during the rainy months (July to October) and form direct connections with the lake during the rainy season through fringes. Therefore, the aim of this study was to investigate whether *Labeobarbus* species and *C. gariepinus* use these wetlands as spawning and/or nursery habitats.

## 2. Methods

### 2.1. Description of Study Area

Lake Tana, Ethiopia's largest lake and the source of Blue Nile River, has a surface area of *ca*. 3200 km^2^. It is situated in the northwestern highlands at an altitude of approximately 1800 m. It is a shallow (maximum depth 14 m, mean 8 m) lake. More than 60 small seasonal tributaries and seven perennial rivers (Gumara, Ribb, Megech, Gelgel Abbay, Gelda, Arno-Garno, and Dirma) feed the lake [17]. The only outflowing river is the Blue Nile; however, the ichthyofauna is isolated from the lower Blue Nile by a 40 m waterfall located 30 km from Lake Tana. Fogera and Dembia floodplains are the largest wetlands of the country and border Lake Tana in the eastern and northern parts, respectively. Welala and Shesher wetlands (Figure 1) are located in the Fogera floodplain.

Fogera *Woreda *(district) is one of the ten Woredas bordering Lake Tana and is found in South Gondar Administrative Zone. It is situated at 11°58′00′′ N latitude and 37°41′00′′ E longitude [18]. Woreta, capital of the Fogera Woreda is found 620 km from Ethiopia's capital city, Addis Ababa and 55 km from Bahir Dar, the regional capital, (Figure 1). Ribb River originates from Gunna Mountains, at an altitude of above 3000 m and has the length of *ca*. 130 km and drainage area of about 1790 km^2^ [21]. In its lower and middle reaches, the river flows over the extensive alluvial Fogera Floodplains. The river meanders and flows slowly over this floodplain, and this resulted in river channel deposition and overflowing of riverbanks and charging water to Welala and Shesher during the rainy season.

 According to the information from the local people, Shesher dries usually in February or March; whereas, Welala dries in April or May. In some years, when there is high overflow from Ribb River, Welala never dries throughout the year (personal communication with the local people). This is because Welala is smaller in size and deeper (maximum depth 2.5 m in the rainy season) as compared to Shesher which is wider and shallower (maximum depth 1.75 m in the rainy season). Location, distance from the lake, elevation and bottom types of the sampling sites are summarized in Table 1. Coordinates and elevations were assessed with a GPS. The bottoms of these two wetlands are muddy (Table 1) and nowadays, during the post rainy season, the local farmers drain the water by digging canals from these two wetlands to get fertile land (the muddy bottom) for crop production [18].

**Figure 2:**
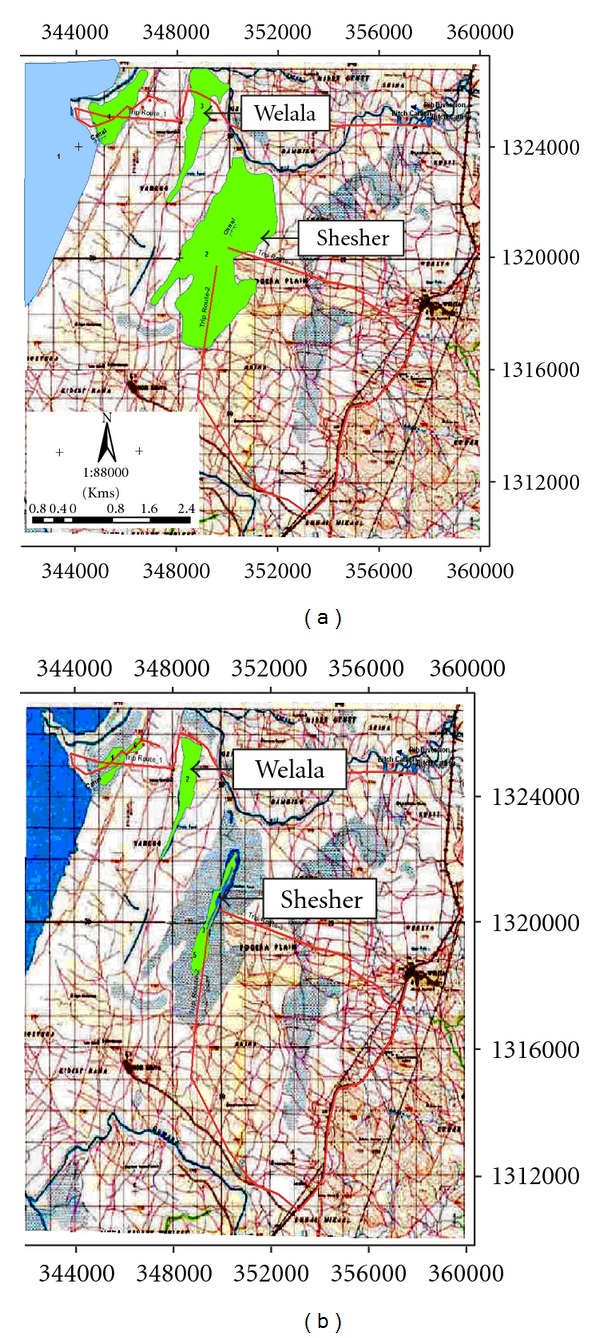
Extent of Shesher and Welala wetlands in 1987 (a) and their current (2008) extent (b) (after Nemomissa [19]).

The drastic changes in the areas of Shesher and Welala wetlands in the last two decades are shown in Figure 2. In 1987, the total surface area of Shesher and Welala was *ca.* 1557 and 298 hectares, respectively (Figure 2(a)) [19]. Whereas, in 2008 the surface area of Shesher shrunk to 136 hectares (91% shrinkage) and Welala shrunk to 159 hectares (47% shrinkage) (Figure 2(b)) [19]. These wetlands are shrinking at an alarming rate, mainly because of unsustainable farming practices by the local inhabitants [18]. The local farmers drain the water of these wetlands to expand their farmland and pump water for irrigation. The large irrigation dam under construction on Ribb River is another potential threat. This dam prevents overflow from Ribb River into the wetlands and disrupts the connection with Lake Tana which is vital for the survival of these wetlands [18–20].

### 2.2. Sampling

Sampling took place from July 2010 to October 2010. Three sampling sites were selected in each wetland, two shore sites and one site at the middle. In the shore sites, gillnets were set at the mouth of the inflow from Ribb River overflow and at the outlets to Ribb River main channel. The outflow from Ribb first enters to Shesher, from the north, and when Shesher is filled, it overflows to Welala, and finally to Lake Tana (Figure 1). Besides Welala and Shesher wetlands, samples were collected at Ribb River mouth. Fish and physicochemical parameters were collected nine times at the seven selected sites, once in July (third week), three times in August (first, second, and fourth weeks), three times in September (first, third, and fourth weeks) and twice in October (first and fourth weeks).

### 2.3. Physicochemical Parameter Measurements

At all the sampling sites, observation of bottom type and measurements of depth and Secchi depth (using 30 cm diameter Secchi-disk) were taken. Similarly, dissolved oxygen, temperature, and pH were measured (using probes) at the surfaces of all sampling sites and at all times, in the morning immediately after overnight gillnet catch collection.

### 2.4. Fish Collection

Multifilament gillnets (6, 8, 10, 12, and 14 cm stretched mesh size) with a panel length of 100 m and depth of 1.5 m were used. Gillnets were set usually at 6 : 00 PM, and catches were collected in the following morning at about 6 : 00 AM. All of the fishes caught were identified to species level with immediate inspection (for *C. gariepinus*) and with the help of identification key developed by Nagelkerke and Sibbing [22] for *Labeobarbus* species. After identification to the species level, each fish was dissected; the gonads were examined visually and sexed. The gonad maturity stage of each specimen of *Labeobarbus* species was determined visually, using the key developed by Nagelkerke [17], but for *C. gariepinus* the gonad maturity stage was determined according to Wudneh [8].

### 2.5. Data Processing

All the statistical computations were done using Minitab version 14 and SPSS version 11 software. Pairwise comparison of dissolved oxygen content (mgL^−1^), temperature (°C) and vertical transparency or Secchi depth (cm) of the seven sampling sites were compared through one-way analysis of variance (one-way ANOVA) followed by Bonferroni's post hoc tests for multiple comparisons if significant variance was evident. One-way ANOVA was also used to investigate temporal segregation of *Labeobarbus* species aggregating at Ribb River mouth and *C. gariepinus* spawning in Shesher and Welala wetlands. Only fish with ripe and spent gonads were considered for temporal segregation analysis as reproductively immature fishes would not be expected to show temporal variation in aggregation and migration. Catch per unit of effort (CpUE) was defined number of fish per overnight gillnet setting.

## 3. Result

### 3.1. Physicochemical Parameters

There was a highly significant overall variation on dissolved oxygen (*F*_(6,56)_ = 33.85; *P* < 0.001) but not in temperature (*F*_(6,56)_ = 2.15; *P* > 0.05), pH (*F*_(6,  56)_ = 0.83; *P* > 0.05), and vertical transparency (*F*_(6,  56)_ = 0.47; *P* > 0.05) among the sampling sites (Tables 2 and 3). All the sampling sites in Shesher and Ribb River mouth have significantly higher dissolved oxygen concentration (*P* < 0.001; Table 3) than the sites in Welala. The pairwise comparison also showed that the sampling sites of Shesher, except site II, have higher dissolved oxygen concentration (*P* < 0.001) than Ribb River mouth (Table 3). The average dissolved oxygen concentration of Shesher (when the three sites pooled), 6.13 ± 0.22, is higher than Welala, 5.14 ± 0.06 (Table 2).

### 3.2. Fish Species Composition and Abundance

**Figure 3:**
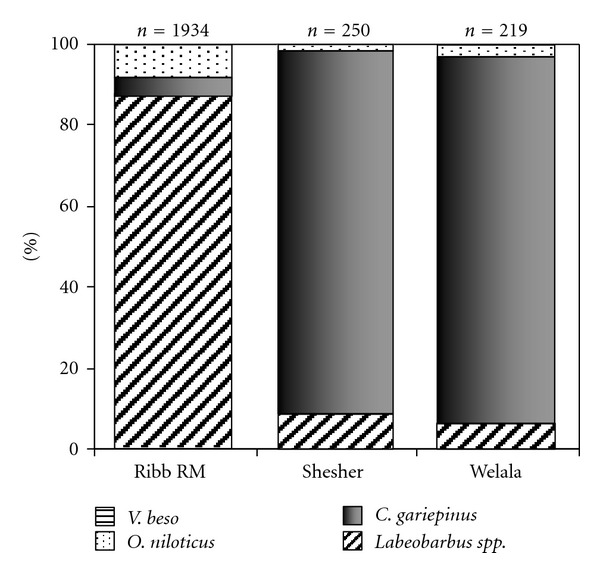
Percentage composition of fish collected at Ribb River mouth and its associated wetlands: Shesher and Welala. Data from all sites (for Shesher and Welala) and months pooled. “*n*” refers to the number of fish.

A total of 2403 fish specimens were collected from Ribb River mouth and Shesher and Welala floodplain wetlands. From this catch, 1725 (72%) specimens were contributed by *Labeobarbus* species, followed by *C. gariepinus* (21%, 506 specimens), *Oreochromis niloticus* (7.1%, 170 specimens) and *Varicorhinus beso* (with two specimens only). *Clarias gariepinus* and *Labeobarbus* spp. were dominant at the two wetlands and Ribb River mouth, respectively (Figure 3).

**Figure 4:**
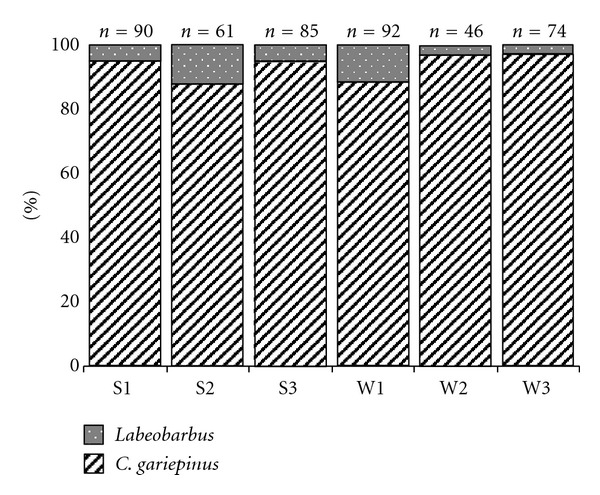
Percentage contribution of *Labeobarbus* spp. and *C. gariepinus* collected from the six different sampling sites of Shesher and Welala wetlands. Pooled data collected from July to October. “S” and “W” stand for Shesher and Welala, respectively. “*n*”, number of specimens.

A total of 469 (250 and 219 from Shesher and Welala, resp.) fish specimens were collected from all the sampling sites of Shesher and Welala wetlands. *Clarias gariepinus* was the most numerous (Figure 4) fish species and contributed about 93% (421 specimens) of the total catch from all sampling sites of the two wetlands. However, *Labeobarbus* species were incidentally caught at Shesher and Welala wetlands (Figure 4). Only 27 (6.2% of the total wetland catch) specimens of *Labeobarbus* were collected from the wetlands. The* Labeobarbus *spp. collected from the two wetlands include *L. acutirostris* (1 specimen), *L. brevicephalus* (8 specimens), *L. megastoma* (3 specimens), *L. intermedius* (14 specimens), and *L. tsanensis* (1 specimen). Together with *C. gariepinus* and *Labeobarbus* spp., only ten specimens of* O. niloticus* were collected from the two wetlands.

From the 15 endemic species of *Labeobarbus* described in Lake Tana, 13 species were collected from Ribb River mouth. However, from these 13 *Labeobarbus* species, only six species (Table 4) were the most dominant contributing nearly 95% (1595 specimens) of the total *Labeobarbus* catch from Ribb River mouth. Seven species (*L. acutirostris, L. crassibarbis, L. gorgorensis, L. longissimus, L. macrophthalmus*, *L. nedgia*, and* L. surkis*) were incidentally captured and contributed less than 8% of the *Labeobarbus* catch from Ribb River mouth. Two species, *L. dainellii* and *L. gorguari*, were totally missing from the catches of the river mouth.

### 3.3. Gonad Maturity Stages

**Figure 5:**
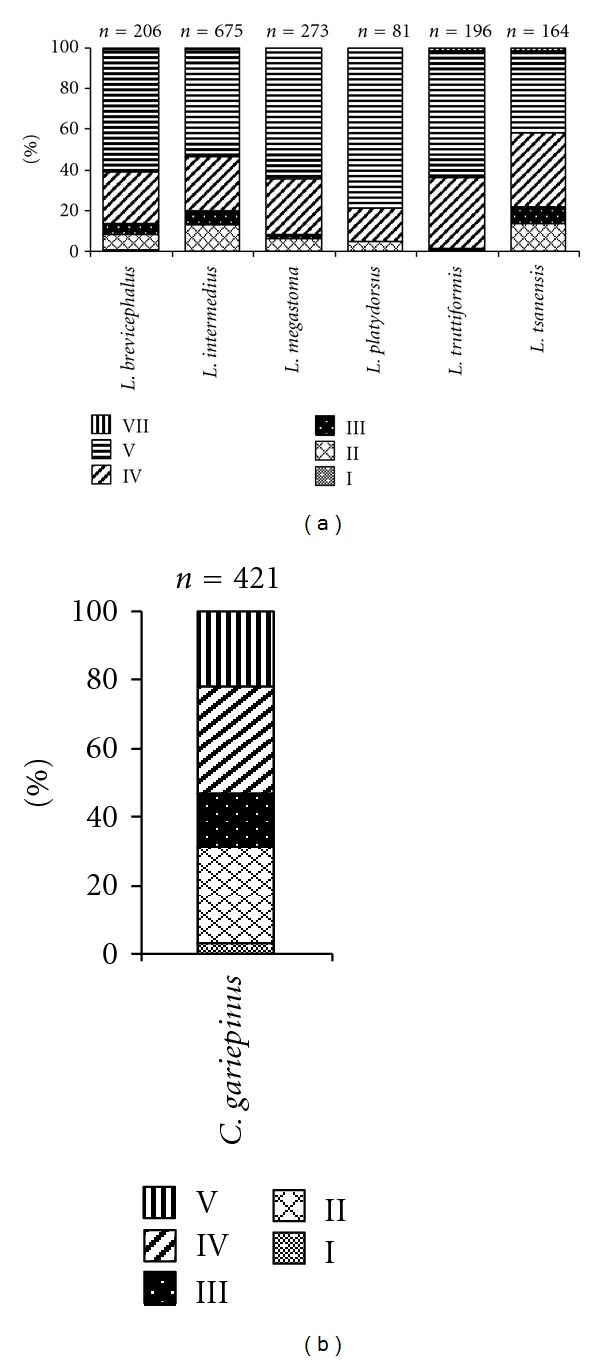
Gonad stages of immature (gonad stages I–III), ripe (gonad stages IV and V) and spent (gonad stage VII) of the six most abundant *Labeobarbus* species aggregating at Ribb River mouth (a), and *C. gariepinus* collected from Shesher and Welala wetlands (b). Note that gonad maturity stage V is ripe for *Labeobarbus* spp., but spent for *C. gariepinus*.

From the total of 1668 specimens of the six *Labeobarbus* species collected from Ribb River mouth, only 101 (6%) were immature (gonad stages II and III); whereas 1563 (93%) specimens were ripe (gonad stages IV and V), and 14 (1%) were spent (gonad stage VII) (Figure 5(a)). However, no running (gonad stage VI) *Labeobarbus* specimen was collected at the river mouth (Figure 5(a)). However, from the total of 88 specimens of *C. gariepinus* collected from Ribb River mouth only 11 (12.5%) were ripe or spawning (gonad stage IV), 77 (87.5%) were immature (gonad stage I–III) and only one specimen was spent (gonad stage V). Of the gonads of *C. gariepinus* collected from Shesher and Welala wetlands, 133 (32%) were ripe, 197 (46.5%) were immature, and 91 (22.5%) were spent (Figure 5(b)).

### 3.4. CpUE and Temporal Segregation

**Figure 6:**
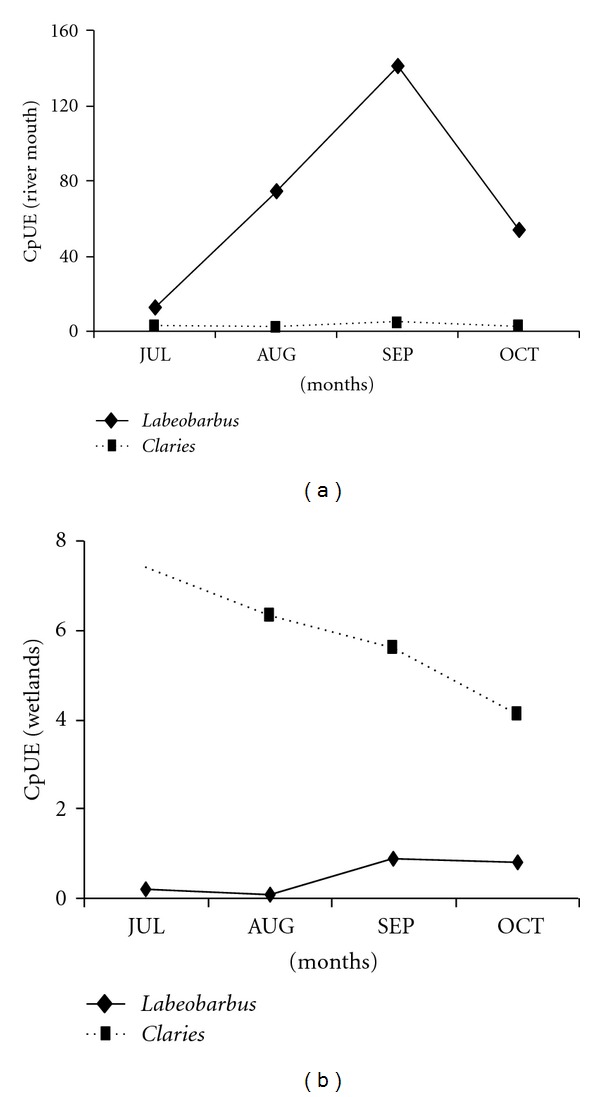
CpUE of *Labeobarbus* and *Clarias* at Ribb River mouth (a) and in Shesher and Welala Wetlands (b).

Collectively, the peak CpUE value for the six *Labeobarbus* species aggregating at Ribb River mouth was observed in September (Figure 6(a)). However, the peak CpUE for *C. gariepinus* collected from Shesher and Welala wetlands was in July (Figure 6(b)), and the slope of the graph remained negative for the whole study period.

**Figure 7:**
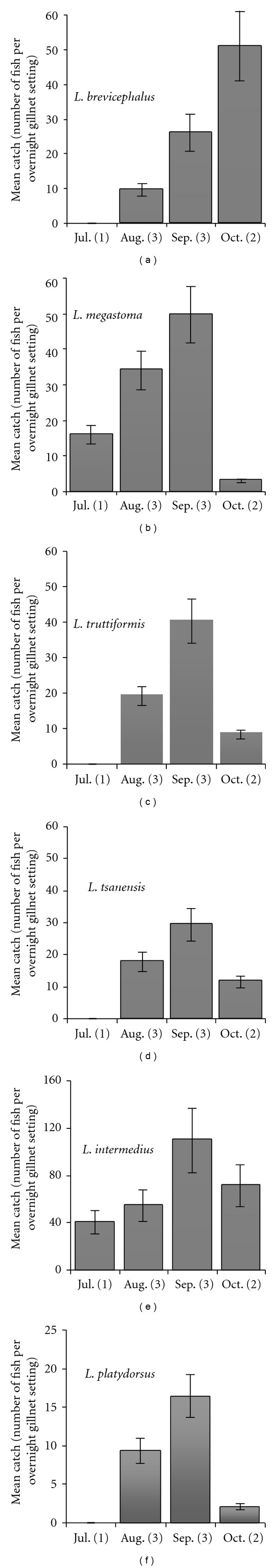
Mean abundance (number of fish per overnight gillnet setting) with 95% confidence limits (CL) of the six *Labeobarbus* species aggregating at Ribb River mouth during the spawning months (July to October).

The monthly catches of the six *Labeobarbus* species aggregating at Ribb River mouth, except *L. intermedius*, showed significant temporal segregation (*P* < 0.05; Table 5). Significant temporal segregation (*P* < 0.05; Table 5) was also evident for *C. gariepinus* spawning at Shesher and Welala wetlands. The details of temporal segregation trends and apparent overlaps among the six *Labeobarbus* species aggregating at Ribb River mouth is shown in Figure 7. *Labeobarbus megastoma* and *L. intermedius *were apparently the first to appear at Ribb River mouth for prespawning aggregation (during July). The peak CpUE for these two species was in September (Figure 7), but it declined from August to October. Whereas, a reverse trend was observed for *L. brevicephalus*, CpUE increases from August to October. The other three species: *L. platydorsus, L. truttiformis, *and* L. tsanensis* aggregate during August and September, but their CpUE declined sharply during October.

## 4. Discussion

The water temperature and pH values obtained in the present study (Table 2) from Shesher and Welala wetlands lie within the same range as Lake Tana's [23]. This is due to the fact that these wetlands have hydrological connections with Lake Tana. Dissolved oxygen was significantly higher (*P* < 0.05) in Shesher than in Welala and Ribb River mouth sampling sites. This is probably due to high mixing by wind, since Shesher is shallow and has no shore vegetation cover. Similarly, high dissolved oxygen concentration was obtained in Shesher by Atnafu et al. [18]. However, as compared to the *Labeobarbus* spawning streams in Gumara [9] and Megech [15], tributaries of Lake Tana, these two wetlands have lower dissolved oxygen concentration, high turbidity, and lack gravel substrate.

 Prespawning aggregations and upstream migrations of *Labeobarbus* species to the tributary rivers of Lake Tana was intensively studied in the last two decades [9, 12–16, 24–26]. These studies showed that seven species of *Labeobarbus*, after making brief prespawning aggregations at the river mouths, migrate more than 50 km up rivers and spawn in clear, fast flowing, well-oxygenated, and gravel bed small streams. Almost all African *Labeobarbus*, whether lake dwelling or riverine, require these conditions in their spawning grounds [3]. However, unlike the other African *Labeobarbus*, eight of the 15 species in Lake Tana, are absent in all the seven perennial tributaries. The most acceptable assumption is that like many other cyprinid genera, the eight missing (species not found to spawn in river tributaries) *Labeobarbus* species most probably breed in the lake and adjacent floodplain wetlands [14]. The use of marginal vegetation of the lake's shore and adjacent floodplain wetlands shelters from predators and provides high densities of prey for larvae and juveniles [27].

Contrary to the above assumption, in the present study, these missing *Labeobarbus* species (*L. nedgia, L. dainellii, L. gorguari, L. longissimus, L. intermedius, L. gorgorensis, L. surkis*, and *L. crassibarbis*) were not found to spawn in the most prominent adjacent floodplain wetlands, Shesher and Welala. This is probably because none of the requirements for *Labeobarbus* spawning were satisfied in the adjacent floodplain wetlands. During the spawning months, these wetlands were so turbid, poorly oxygenated, and the bottom is muddy, instead of gravel (Table 1). Unlike *C. gariepinus* and Nile tilapia, *Labeobarbus* species in Lake Tana are ecologically specialized and highly vulnerable [7]; hence, it is unlikely that the larvae of *Labeobarbus* will survive under these poor spawning ground conditions. Another explanation could be the existence of *C. gariepinus* in mass in these two wetlands (Figures 3 and 4) that predate on fish. The larvae and juveniles of *Labeobarbus* could be preyed upon by *C. gariepinus *[8]; hence, coexistence of *C. gariepinus* and early life stages of *Labeobarbus* in this relatively small wetlands would result in high mortality rates of juvenile *Labeobarbus*.

 In the present study, a large number of *C. gariepinus* were collected from Shesher and Welala Wetlands. *Clarias gariepinus* is well adapted to the environmental conditions of the wetlands, it is highly tolerant to low oxygen levels and high turbidity. Although the CpUE of *C. gariepinus* during our sampling declines from July to October, the presence of a relatively high percentage of spent implies that a substantial proportion of the fish may not drift back to the lake, rather they spend the rest of their life, after spawning, in these wetlands. The presence of a relatively high proportion of immature individuals (46.5%) in our catch indicates the presence of feeding migration of *C. gariepinus* to these wetlands as well. It was indicated by the local people that they observe mass of juveniles of *C. gariepinus* in these wetlands during the postrainy season (end of October and November). Most of the dry *C. gariepinus* exported to neighbouring countries such as Sudan is obtained from Shesher and Welala wetlands [11], and intensive fishing activities by local people using seine nets takes place in February and March. Probably, most of the juveniles of *C. gariepinus* in the nearby shallow inundated floodplains move to these wetlands for growth, since these wetlands get dry usually in April or May. What the local people intensively catch are those immature feeding migrants that stayed in these wetlands, young of the year (juveniles), and those spent *C. gariepinus* that remained in the wetlands.

 Unlike *C. gariepinus*, only few specimens of *Labeobarbus* species aggregating at Ribb River mouth have reproductively immature gonads, more than 80% were ripe. However, no running (gonad stage VII; shedding eggs and sperm) *Labeobarbus* was collected from Ribb River mouth. This supports the suggestion made by Palstra et al. [9] which states that, if spawning maturity is only reached when the fish arrives at its spawning ground (more than 30 km up rivers), there could be a fine-tuning between homing and gonad development. Figure 7 shows the details of temporal segregation and overlapping during the spawning months (July to October) among the six *Labeobarbus* species aggregating at Ribb river mouth. All these six *Labeobarbus*, except *L. intermedius*, were temporally segregated in their spawning aggregation at Ribb River mouth in the four spawning months. *Labeobarbus intermedius* is the only *Labeobarbus* species in Lake Tana that spawns throughout the year, and ripe individuals are always common in the river mouths [14]. *Labeobarbus megastoma* migrates from the lake to the river mouth, starting in July, and the CpUE declined after September. Three species, *L. truttiformis, L. platydorsus* and *L. tsanensis*, start to aggregate in August and CpUE reached peak in September and then started to decline. The last species to aggregate was *L. brevicephalus*, its peak is in October. Strong temporal segregation during prespawning aggregation at the river mouths among the migratory riverine *Labeobarbus* species was reported by de Graaf et al. [14]. In addition to the six species found to aggregate at Ribb River mouth in the present study, de Graaf et al. [14] observed two more species, *L. acutirostris *and* L. macrophthalmus* in four tributaries of Tana (Gelgel Abbay, Gelda, Gumara and Ribb) from July to October [14]. Moreover, these two species were also found to migrate more than 30 km in Gumara River upstream [9]. But, similar to the present study, both species did not form prespawning aggregations in other tributaries, Dirma and Megech Rivers [25] and Arno-Garno River [16]. These irregularities probably originate from the traditional fish migration study methods used (e.g., CpUE data). Other modern fish migration study methods such as radio-tracking may supplement the existing information and clarify the secrecy of the spawning grounds of those eight *Labeobarbus* species not found in the tributaries of rivers.

 The hypothesis that the eight Labeobarbus species that do not migrate to rivers for spawning may spawn in adjacent floodplain wetlands [14] was not supported in the present study. The other possibility is that they may spawn in the rocky shores of Lake Tana [14]. Although the absence of these species in the tributary rivers and adjacent wetlands does not automatically mean they spawn in the lake, lake spawning now seems the best option. However, we again strongly recommend the application of radio-tracking or other dependable methods to investigate the actual spawning place(s) of these eight missing *Labeobarbus* species. Since Lake Tana and its shore wetlands are under heavy human pressure, mapping the spawning habitat is essential to conserve this unique *Labeobarbus* species flock.

## Figures and Tables

**Table 1:** Sampling sites, estimated distance from the lake, coordinates, elevation, and bottom types of the sampling sites.

Sampling site	Estimated distance (km) from the lake	Coordinates	Elevation	Bottom type
Ribb River mouth	—	N 11°59′54.2′′ E 37°33′06.1′′	1788 m	Mud and Silt (mixed)
Welala I	3.5	N 11°58′59.1′′ E 37°36′12.3′′	1789 m	Mud
Welala II	3.5	N 11°58′38.2′′ E 37°36′34.9′′	1789 m	Mud
Welala III	3.5	N 11°58′14.1′′ E 37°36′18.6′′	1789 m	Mud
Shesher I	4	N 11°58′25.3′′ E 37°37′16.4′′	1791 m	Mud
Shesher II	4	N 11°57′01.3′′ E 37°37′27.5′′	1791 m	Mud
Shesher III	4	N 11°56′58.2′′ E 37°37′35.3′′	1791 m	Mud

**Table 2:** Mean ± SE (Standard Error) values of oxygen concentration, temperature, pH, and Sechi-disk depth at the sampling sites. *N* refers to number of samplings.

Site name	*N*	Oxygen (mgL^−1^)	Temp. (°C)	pH	Secchi-disk depth (cm)
Ribb River mouth	9	5.66 ± 0.09	21.2 ± 0.14	6.97 ± 0.03	4.56 ± 1.04
Welala I	9	5.12 ± 0.05	21.5 ± 0.22	7.02 ± 0.03	5.33 ± 0.76
Welala II	9	5.15 ± 0.05	20.9 ± 0.21	7.01 ± 0.01	5.32 ± 0.51
Welala III	9	5.14 ± 0.08	21.1 ± 0.23	6.99 ± 0.10	6.11 ± 0.92
Shesher I	9	6.17 ± 0.37	21.6 ± 0.22	6.96 ± 0.02	6.22 ± 1.06
Shesher II	9	6.02 ± 0.09	21.7 ± 0.24	7.02 ± 0.01	6.11 ± 1.01
Shesher III	9	6.19 ± 0.19	21.4 ± 0.21	7.03 ± 0.05	6.00 ± 0.83

**Table 3:** Pairwise comparison of dissolved oxygen concentrations (mgL^−1^) among sampling sites. Abbreviation used: RM = river mouth.

	Ribb RM	Welala I	Welala II	Welala III	Shesher I	Shesher II	Shesher III
Ribb RM	×						
Welala I	**	×					
Welala II	**	NS	×				
Welala III	**	NS	NS	×			
Shesher I	**	***	***	***	×		
Shesher II	NS	***	***	***	NS	×	
Shesher III	**	***	***	***	NS	NS	×

*P < 0.05; **P < 0.01; ***P < 0.001; NS: not significant; *P* > 0.05.

**Table 4:** The abundance of the six most dominant *Labeobarbus* species collected from Ribb River mouth, and their catch from Shesher and Welala wetlands.

Species	Number of specimens		
	Ribb River mouth	Shesher	Wolala
*L. brevicephalus*	206	10	3
*L. intermedius*	675	7	10
*L. megastoma*	273	2	2
*L. platydorsus*	81	0	0
*L. truttiformis*	196	0	0
*L. tsanensis*	164	1	0

**Table 5:** One-way ANOVA result on the catch data of the six *Labeobarbus* species from Ribb River mouth and *C. gariepinus* from Shesher and Welala wetlands, for differences in the four spawning months (July–October).

Species	Source of variation	MS	*F*_(3,5)_ value	*P*
*L. brevicephalus*	Month	695.19	17.04	**
*L. intermedius*	Month	2216.72	5.24	NS
*L. megastoma*	Month	873.41	65.51	***
*L. platydorsus*	Month	103.69	6.23	*
*L. truttiformis*	Month	589.46	36.02	**
*L. tsanensis*	Month	176.13	8.32	*
*C. gariepinus*	Month	391.54	33.57	**

*P < 0.05; **P < 0.01; ***P < 0.001; NS: not significant, *P* > 0.05.
